# Research on Modeling and Analysis of Generative Conversational System Based on Optimal Joint Structural and Linguistic Model

**DOI:** 10.3390/s19071675

**Published:** 2019-04-08

**Authors:** Yingzhong Tian, Yafei Jia, Long Li, Zongnan Huang, Wenbin Wang

**Affiliations:** 1 School of Mechatronic Engineering and Automation, Shanghai University, Shanghai 200072, China; troytian@shu.edu.cn (Y.T.); 18817606385@163.com (Y.J.); lil@shu.edu.cn (L.L.); zongnan_shl63@163.com (Z.H.); 2 Shanghai Key Laboratory of Intelligent Manufacturing and Robotics, Shanghai 200072, China; 3 School of Mechanical and Electrical Engineering Shenzhen Polytechnic, Shenzhen 518055, China

**Keywords:** generative conversational system, the joint structural and linguistic model, a new convolutional neural network architecture, strengthened attention with intention, foolish punishment mechanism

## Abstract

Generative conversational systems consisting of a neural network-based structural model and a linguistic model have always been considered to be an attractive area. However, conversational systems tend to generate single-turn responses with a lack of diversity and informativeness. For this reason, the conversational system method is further developed by modeling and analyzing the joint structural and linguistic model, as presented in the paper. Firstly, we establish a novel dual-encoder structural model based on the new Convolutional Neural Network architecture and strengthened attention with intention. It is able to effectively extract the features of variable-length sequences and then mine their deep semantic information. Secondly, a linguistic model combining the maximum mutual information with the foolish punishment mechanism is proposed. Thirdly, the conversational system for the joint structural and linguistic model is observed and discussed. Then, to validate the effectiveness of the proposed method, some different models are tested, evaluated and compared with respect to Response Coherence, Response Diversity, Length of Conversation and Human Evaluation. As these comparative results show, the proposed method is able to effectively improve the response quality of the generative conversational system.

## 1. Introduction

Along with the rapid development of artificial intelligence, the use of generative conversational systems based on joint structural and linguistic models is increasingly being observed and is being applied in some interesting robotic cases. Generative conversational systems provide the ability to generate conversational responses actively. Additionally, they are also not limited by conversation content. Implicitly, this provides several benefits for human life, such as in the family environment, hospitals, entertainment venues, etc.

Conversational systems are composed of a neural network-based structural model and a linguistic model. The neural network-based structural model mainly performs feature extraction and semantic understanding on input sequences. In addition, the linguistic model can determine the probability of the existence of the output sequence by determining a probability distribution for an output sequence of length m. The response quality of the system, with respect to aspects such as diversity, informativeness and multi-turns, is greatly influenced by different structural models and linguistic models. However, generative conversational systems, studied by many researchers on the basis of unilateral factors in structural models and linguistic models, are prone to generating responses that lack informativeness, diversity, and multi-turns, such as “I don’t know” and “I have no idea”. Thus, to improve the response quality of the conversational system in those terms, an optimal combination of the improved structural model and enhanced linguistic model is implemented.

As an effort towards improving the response quality, structural models of conversational systems based on Recurrent Neural Networks (RNN) are currently being studied. Meanwhile, with the introduction and wide application of attention models in the conversational field, the semantic understanding of input sequences by conversational systemconversational systems has been greatly improved [1,2]. Attention models can ensure that input tokens are aligned with output tokens, and then distribute attention weights according to the contribution of each token to the input sequence. Therefore, the understanding of deep semantics and the transmission of semantic vectors in the models is of great significance. To increase the diversity of responses, latent variables have also been added to structural models. For example, a standard Gauss latent variable Z is added to the encoder–decoder to increase the response diversity [3]. The latent variable hierarchical recurrent encoder–decoder (VHRED) model has been proposed to augment the HRED [4] model with a stochastic latent variable at the utterance level [5]. The model was trained by maximizing a variational lower-bound on the log likelihood so that the model could facilitate the generation of diverse responses while maintaining the conversational state. Meanwhile, the hierarchical recurrent encoder–decoder with separated context (SPHRED) [6] added a conditional variational framework to the HRED model for response generation. Thereby, responses were able to be generated in accordance with the specific attributes by the model. Similarly, topic information was added to structural models in order to increase the response informativeness. By adding the topic model to the sequence-to-sequence model [7], the topic-aware sequence-to-sequence (TA-Seq2Seq) is able to utilize topics to simulate prior human knowledge, guiding them to form informative and interesting responses in conversation, and leveraging the topic information in generation by a means of a joint attention mechanism and biased generation probability [1]. Moreover, a deep Long Short-Term Memory network (LSTM) model composed of a forward network and a backward network in the input layer was proposed to capture the forward and backward context [8]. The input layer was different from the bidirectional LSTM layer, whose input process was sequential in time, i.e., the input layer reranked the output of the forward network and entered it into another trained network to capture the forward and backward contexts. So far, however, most structural models of conversational systems have been developed on the basis of RNN, whereby the memory of RNN performs very poorly under testing. In addition, structural models showing improvements in terms of joint diversity, informativeness, or multi-turns have rarely been reported. Meanwhile, Convolution operations have strong advantages in speech recognition and machine translation [9,10,11,12,13], which can capture long-term context. A gated convolutional network model, which could increase network layers and control information flows through convolution and gating operations, has been proposed for language modeling in machine translation [9]. A neural machine translation model consisting of the single-layer convolutional encoder networks and LSTM decoder has also been proposed in machine translation [14]. The single-layer convolution encoder networks were able to increase network layers using stacked convolution layers, and the performance of the model was greatly improved. Therefore, in this paper, convolution operations are introduced to the structural model of generative conversational systems. However, edge words of variable-length input sequence are easily lost when subjected to convolution. Additionally, because of over-fitting, the number of convolution layers is limited. According to the structural model described, a method using a Multi-hierarchical Convolutional Neural Network (CNN) architecture is proposed to deal with variable-length input sequences. In addition, a novel dual-encoder structural model based on the new CNN and strengthened attention with intention is established in order to improve the response quality in many respects.

Common and foolish responses are often generated by the prediction of responses with the general statistical linguistic model in conversational system. Meanwhile, linguistic models based on Maximum Mutual Information (MMI), Mutual Information (MI), Pointwise Mutual Information (PMI) and Term Frequency–Inverse Document Frequency (TF-IDF) are also derived to increase the coherence between the input sequence and system response. For example, responses that enjoy unconditionally high probability, as well as biases towards responses that were specific to the given input, could be avoided by the linguistic models based on MMI [15]. The responses that enjoy high probability but were ungrammatical or incoherent could be avoided by the linguistic models based on MI [16]. The nonspecific responses could be avoided by the linguistic models that incorporated the TF-IDF term [2]. Similarly, the linguistic models based on PMI were able to predict a noun as a keyword reflecting the main gist of the response in order to generate a response containing the given keyword [8]. These studies of the coherence between the input sequence and system response can increase the informativeness to some extent, but more foolish responses are still unavoidable in testing. Therefore, a linguistic model based on MMI and a foolish punishment mechanism is proposed. 

To comprehensively improve the response quality of the conversational system with respect to the aspects of the structural model and the linguistic model, the attention with intention-based structural model and TF-IDF-based linguistic model were combined [2]. The joint model firstly modeled intention across turns using RNN, and then incorporated an attention model that was conditional on the representation of intention. It subsequently avoided generating non-specific responses by incorporating an IDF term in the linguistic model. A structural model based on forward and backward neural networks and a linguistic model based on PMI were also combined [8]. The joint model firstly used PMI to predict a keyword, then generated a response containing the keyword using the structural model. These joint models improved the informativeness of system responses by combining the developed structural model and linguistic model. Therefore, in order to improve the response quality of conversational systems in terms of diversity, informativeness and multi-turns, a novel joint model is established in this paper, which combines the dual-encoder structural model with the linguistic model. The theoretical model is also proven to be effective by comparison with an experiment.

To address the problem of the lack of diversity, informativeness and multi-turns, a joint model is presented in the paper. In Section 2, a novel dual-encoder structural model based on the new CNN and strengthened attention with intention is established. In Section 3, the linguistic model based on MMI and the foolish punishment mechanism is established. In Section 4, the experiments on generative conversational system based on the joint structural and linguistic model are built. In Section 5, comparisons are drawn between the joint model and baseline models.

## 2. Model Architecture

**Figure 1:**
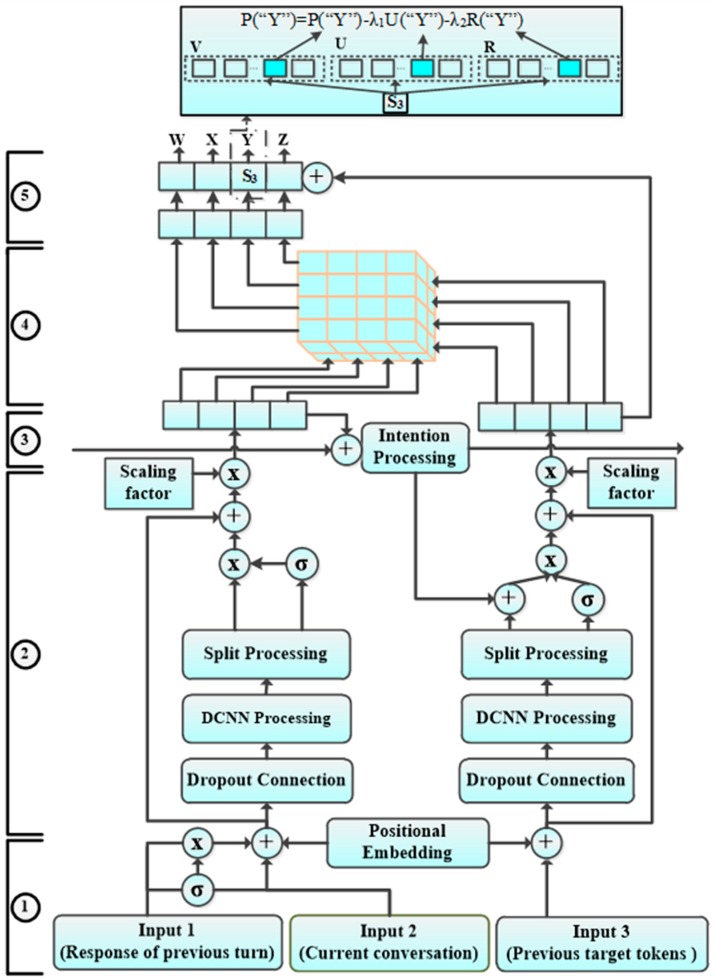
The dual-encoder structural model based on the new CNN and strengthened attention with intention. ① is the input pre-processing layer; ② is the dual-encoder layer (Encoder 1: left; Encoder 2: right); ③ is the conversational intention layer; ④ is the strengthened attention layer; ⑤ is the fully connected layers.

In this section, a novel dual-encoder model structure based on the new CNN and strengthened attention with intention (SAWI-DCNN) is proposed, where CNN, rather than RNN, can be used to obtain the long-term context. First, the pre-processed input sequences are processed in encoder 1, as shown in Figure 1. Meanwhile, previous target tokens are processed in encoder 2. Second, the output sequence of encoder 1 distributes attention at the strengthened attention layer, where the distribution of attention is affected by the state of encoder 2, including conversational intention [2,17,18]. Finally, the output sequence of the attention distribution and encoder 2 is iterated to generate the predicted target token at the fully connected layer. 

### 2.1. Input Pre-Processing

The input sequence $X^{\left(k\right)}=\{x_{1}^{\left(k\right)},x_{2}^{\left(k\right)},x_{3}^{\left(k\right)},\cdots ,x_{m}^{\left(k\right)}\}$ with the length m is transformed into embedded vectors $E^{\left(k\right)}=\{e_{1}^{\left(k\right)},e_{2}^{\left(k\right)},e_{3}^{\left(k\right)},\cdots ,e_{m}^{\left(k\right)}\}$ by an embedded matrix $D\in R^{V\times f}$, where $e_{m}^{\left(k\right)}\in R^{f}$ represents the embedding vector at the position m during the k turn conversation. The features and deep semantics of embedded vectors $E^{\left(k\right)}$ are extracted and mined in the conversational system model. However, deeply hidden semantics can only be excavated by the system with difficulty when context is discarded in different interactions. Conversely, too much noise is brought into the conversational system when the context is included in its entirety. Thus, the input regarding the response of the previous turn is controlled in encoder 1 in order to increase the perception of the conversational environment and improve the interaction turns. The updated input vectors $E_{\mathrm{new}}^{\left(k\right)}$ can be defined as

(1) $$E_{\mathrm{new}}^{\left(k\right)}{=E}^{\left(k\right)}+f\left(\bar{e_{E}^{\left(k\right)}},\bar{e_{Y}^{\left(k-1\right)}}\right)$$

where $\bar{e_{E}^{\left(k\right)}}$,$\bar{e_{Y}^{\left(k-1\right)}}\in R^{f}$ are the sentence-level embedded vectors [19] of the input sequence of the current turn k and the output sequence of the previous turn k − 1, respectively. Note that the result of f(·) is a biased vector, which is able to control the input generated by the previous output sequence. $f(\bar{e_{E}^{\left(k\right)}},\bar{e_{Y}^{\left(k-1\right)}})$ is expressed as

(2) $$f\left(\bar{e_{E}^{\left(k\right)}},\bar{e_{Y}^{\left(k-1\right)}}\right)=\cos(\bar{e_{E}^{\left(k\right)}},\bar{e_{Y}^{\left(k-1\right)}})\cdot \bar{e_{Y}^{\left(k-1\right)}}$$

Thus, Equation (1) can be rewritten as

(3) $$E_{\mathrm{new}}^{\left(k\right)}{=E}^{\left(k\right)}+\cos(\frac{\sum {}_{m\in E^{\left(k\right)}}e_{m}}{\left|\sum {}_{m^{\prime }\in E^{\left(k\right)}}e_{m^{\prime }}\right|},\frac{\sum {}_{m\in Y^{\left(k-1\right)}}e_{m}}{\left|\sum {}_{m^{\prime }\in Y^{\left(k-1\right)}}e_{m^{\prime }}\right|})\times \frac{\sum {}_{m\in Y^{\left(k-1\right)}}e_{m}}{\left|\sum {}_{m^{\prime }\in Y^{\left(k-1\right)}}e_{m^{\prime }}\right|}$$

When embedded vectors are input into CNN, multiple vectors are convoluted simultaneously by convolution kernels. In addition, the sense of order of vectors decreases with the increase of the convolution layer. For this reason, the absolute position is embedded in the input sequence in order to increase the temporal order of vectors and enable the model to perceive which part of the input sequence is being processed. The joint embedding vector is expressed as

(4) $$S^{\left(k\right)}{=E}_{\mathrm{new}}^{\left(k\right)}{+P}^{\left(k\right)}{=\{e}_{1}^{\left(k\right)}{+p}_{1}^{\left(k\right)}{,e}_{2}^{\left(k\right)}{+p}_{2}^{\left(k\right)}{,e}_{3}^{\left(k\right)}{+p}_{3}^{\left(k\right)},\cdots {,e}_{m}^{\left(k\right)}{+p}_{m}^{\left(k\right)}\}$$

where $S^{\left(k\right)}\in R^{m\times f}$ is the joint input vectors and $P^{\left(k\right)}{=\{p}_{1}^{\left(k\right)}{,p}_{2}^{\left(k\right)}{,p}_{3}^{\left(k\right)},\cdots {,p}_{m}^{\left(k\right)}\}$ is the position vectors for $p_{m}^{\left(k\right)}\in R^{f}$.

### 2.2. Dual-Encoder

The dual-encoder consists of stacked convolution blocks, which include the new CNN, Gated Linear Units (GLU) [9], Residual connections [20], and scaling factors. The outputs of the convolution blocks are represented as $h_{S}^{\left(k,l\right)}{=[h}_{s1}^{\left(k,l\right)}h_{s2}^{\left(k,l\right)}h_{s3}^{\left(k,l\right)}\cdots h_{\mathrm{sm}}^{\left(k,l\right)}]\in R^{\mathrm{mf}}$ and $h_{D}^{\left(k,l\right)}=[h_{d1}^{\left(k,l\right)}h_{d2}^{\left(k,l\right)}h_{d3}^{\left(k,l\right)}\cdots h_{\mathrm{dm}}^{\left(k,l\right)}]\in R^{\mathrm{mf}}$ in encoder 1 and encoder 2, respectively. Each convolution kernel is parameterized as $W\in R^{w}$, $b_{w}\in R$ in the new CNN. In addition, the input vectors S^(k)^ are mapped to the output vectors $Y\in R^{2m\times f}$ through the new CNN, in which the output vectors have twice the dimensionality of the input vectors.

The information flows of the output $Y=[A,B]\in R^{2m\times f}$ of the new CNN can be controlled by GLU, which provides a linear path for information gradient flows and solves the gradient problem caused by nonlinear gating. Thus, the gated linear unit is added to the convolution blocks.

(5) f(A,B)=A⊗σ(B)

where A and B are a nonlinear input, $A,B\in R^{m\times f}$; ⊗ refers to the point multiplication operation; the dimension of the output $f(\cdot )\in R^{m\times f}$ is half the size of Y; the information flow A related to the current context is controlled by the gates σ(B).

Meanwhile, in order to enable the conversational system to further mine deeply semantic information in a conversational environment, the conversational intention vector $Z^{\left(k\right)}\in R^{f}$ is added to encoder 2 as a bias of the convolution output.

(6) $$f\left({A,B,Z}^{\left(k\right)}\right){=(A+Z}^{\left(k\right)})⊗\sigma (B)$$

Residual connections from the input of each convolution block to the linear gating output are added to avoid degradation caused by network depth. In addition, the scaling factors μ are also added to the convolution blocks to preserve the input variance at the beginning of training. Thus, the output of the convolution block can be expressed as

(7) $$h_{S}^{\left(k,l\right)}=\mu \times (f\left(A⊗\sigma \left(B\right)\right){+h}_{S}^{\left(k,l-1\right)})$$

(8) $$h_{D}^{\left(k,l\right)}=\mu \times (f\left({(A+Z}^{\left(k\right)})⊗\sigma \left(B\right)\right){+h}_{D}^{\left(k,l-1\right)})$$

where $h_{S}^{\left(k,l-1\right)}$ and $h_{D}^{\left(k,l-1\right)}$ are the outputs of l − 1th convolution block in encoder 1 and encoder 2, respectively; meanwhile, the scaling factor μ is a hyper parameter that satisfies $\mu =\sqrt{0.5}$.

In the test, the distribution of the target sequence token is predicted at the top level of the fully connected layers through the linguistic model based on MMI and the foolish punishment, as shown in Section 3.

### 2.3. 1-D Dynamic Convolutional Neural Networks (DCNN)

**Figure 2:**
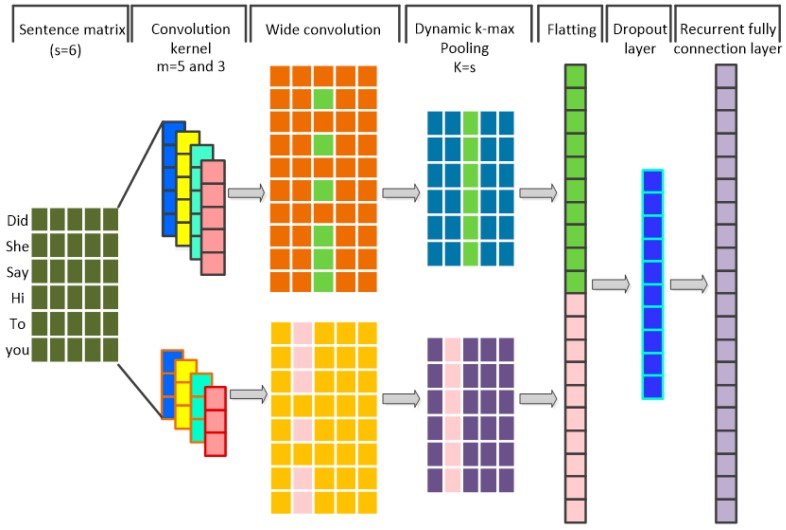
The structure of Dynamic Convolutional Neural Networks.

Since the dimension of input vectors is reduced when the convolution and pooling of the vectors are performed by CNN, it is difficult to increase the number of CNN layers when dealing with variable-length vectors of the input sequence. Therefore, a new Convolutional Neural Network architecture, consisting of a one-dimensional Wide Convolution layer, a dynamic k-max pooling layer, a flatting layer, a dropout layer, and a recurrent fully connected layer, is proposed. As shown in Figure 2, one-dimensional Wide Convolution Operations are adopted [21]. This aims to ensure that the vectors of the whole variable-length input sequence containing the edge words are convoluted by convolution kernels, generating a non-empty feature map c. The two-channel and multi-convolution kernels are used for convolution in order to improve convolution speed and obtain more features. This is initiated by defining the convolution kernels width with one dimension. In addition, the dropout layer is used for regularization. This aims to prevent the occurrence of over-fitting and divergence of the prediction. Meanwhile, in order to align the variable-length vector of both the input and output sequences, a recurrent fully connected layer is proposed. The recurrent fully connected layer is similar to the fully connected layer in RNN. In addition, the dimension of the recurrent fully connected layer is defined as an integer multiple of the input token vector. Finally, the output is generated by sliding the fully connected layer.

(9) $$c_{\mathrm{ij}}=f(\sum M^{T}⊗S_{i-m+1:i,j}{+b}_{m})$$

where $M\in R^{m}$ is a convolution kernel; $b_{m}\in R$ is a bias; $S\in R^{s\times f}$ is the input vectors; $c\in R^{\left(s+m-1\right)\times f}$ is a feature map trough convolution operation and f(·) is an activation function. 

The dimensions of vectors after wide convolution are variable with the varying lengths of different input sequences. The edge vectors are expanded by means of zero filling when the vectors of the input sequence are convoluted by convolution kernels. Thus, the dimension of the convoluted feature map is larger than the input sequence vectors. The one-dimensional dynamic k-max pooling process is defined in order to align the output vector state with the input sequence vectors at each moment. The pooling parameter is defined as

K = s(10)

where s is the length of the input sequence.

The pooled feature map of the single channel convolution and pooling operations is represented as $C_{\max}\in R^{s\times f}$, where the sequences of the feature map values are related to the source and the subscripts are arranged from small to large.

(11) $$C_{\max}=\left[\begin{array}{c} {\mathrm{k-max}(c}_{:,1})\\ \vdots \\ {\mathrm{k-max}(c}_{:,f}) \end{array}\right]$$

### 2.4. Centralizing Intention

The attention weights of the input sequence can be distributed each time using attention models. In addition, according to the attention distribution, the semantic information of the input sequence can be further understood by the conversational system. The attention distribution of the encoder 1 output state can be affected not only by the previous output state of encoder 2, but also by the conversation intention [2,7,16], just like a human being. Conversation intention can represent the conversation context and the primary motivation of the conversation. However, the role of conversation intention in conversation responses is not immediately obvious. This is mainly influenced by the desire that the additional noise have no contribution to the distribution of attention. Thus, to reduce the redundancy of intention caused by the increase in conversation turns, a dynamic model of the intention vector is established, and forgetting gates are added to the model. Hence, the final dynamic model of the intention vector can be expressed as

(12) $$Z^{\left(k\right)}=\tan h(f_{t}\cdot Z^{\left(k-1\right)}+\bar{h_{S}^{\left(k,\mathrm{top}\right)}})$$

where $Z^{\left(k\right)}\in R^{f}$ is an intention vector of the *k*-th turn; tanh(·) refers to the tanh operation and f_t_ is a forgetting gate that can control the previous intention. $f_{t}\in R^{f\times f}$ is expressed as

(13) $$f_{t}=\sigma (\bar{h_{S}^{\left(k,\mathrm{top}\right)}}W_{t}{+b}_{t})$$

where $W_{t}\in R^{1\times f}$ is a transformation matrix; $b_{t}\in R^{f\times f}$ is a bias and $\bar{h_{S}^{\left(k,\mathrm{top}\right)}}\in R^{f}$ is a sentence-level vector of the encoder output at the *k*-th turn that can be expressed as

(14) $$\bar{h_{S}^{\left(k,\mathrm{top}\right)}}=\frac{\sum_{w\in S}h_{w}^{\left(k,\mathrm{top}\right)}}{\left|\sum_{w^{\prime }\in S}h_{w^{\prime }}^{\left(k,\mathrm{top}\right)}\right|}$$

where $\bar{h_{w}^{\left(k,\mathrm{top}\right)}}\in h_{S}^{\left(k,l\right)}$ is the output vectors of the top-layer convolution block in encoder 1.

### 2.5. Intensity-Strengthening Attention

Because the attention weights are distributed according to the contribution of each token in the sequence, and the sum of the attention weights is 1, the effect of a single attention [22] becomes weaker and weaker as the input sequence increases in size. Indeed, the distribution of a single attention will be more distracted, and can even reach zero when the input sequence is longer. An intensity-strengthening attention method is proposed in order to address the problem of the small attention distribution and the partial over-distribution.

To preserve more context for the current state of encoder 2, the previous output sequence is convoluted. Thus, the features of the output sequence at the current time are as follows:

(15) $$\bar{h_{\mathrm{Di}}^{\left(k,\mathrm{top}\right)}}=\frac{\sum_{w\in D}h_{w}^{\left(k,\mathrm{top}\right)}}{\left|\sum_{w^{\prime }\in D}h_{w^{\prime }}^{\left(k,\mathrm{top}\right)}\right|}$$

where $h_{w}^{\left(k,\mathrm{top}\right)}\in h_{D}^{\left(k,l\right)}$ is the output vectors of the top-layer convolution block in encoder 2.

The current state of encoder 2 consists of the features of the output sequence and the previously predicted target token $g_{i-1}^{\left(k\right)}$, which are expressed as

(16) $$d_{i}^{\left(k\right)}{=W}_{D}\bar{h_{\mathrm{Di}}^{\left(k,\mathrm{top}\right)}}{+b}_{D}{+g}_{i-1}^{\left(k\right)}$$

The query vector $d_{i}^{\left(k\right)}$ and the key vectors $h_{\mathrm{Sj}}^{\left(k,\mathrm{top}\right)}$ are mapped to different spaces, and the attention $a_{ijh}^{\left(k\right)}$ is calculated by point multiplication.

(17) $$a_{\mathrm{ijh}}^{\left(k\right)}=\frac{{\exp((W}_{h}^{Q}d_{{}_{i}}^{\left(k\right)})\cdot {(W}_{h}^{K}h_{\mathrm{Sj}}^{\left(k,\mathrm{top}\right)}))}{\sum_{t=1}^{m}{((W}_{h}^{Q}d_{{}_{i}}^{\left(k\right)})\cdot {(W}_{h}^{K}h_{\mathrm{St}}^{\left(k,\mathrm{top}\right)}))},\;h=1,\;2,\;3,\;\cdots ,\;h$$

where $W_{h}^{Q}\in R^{\mathrm{dx}\times f}$ and $h_{h}^{K}\in R^{\mathrm{dx}\times f}$ are transformation matrices. Therefore, the input $C_{i}^{\left(k\right)}$ to the connection layer can be expressed as

(18) $$C_{i}^{\left(k\right)}=\sum_{h=1}^{h}\sum_{j=1}^{m}a_{\mathrm{ijh}}{(W}_{h}^{V}h_{\mathrm{Sj}}^{\left(k,\mathrm{top}\right)})$$

where $W_{h}^{V}\in R^{\mathrm{dx}\times f}$ is a transformation matrix.

The overall intensity of attention is enhanced through superimposed attention, which reduces the effects on attention of both distraction and inattention. The output of encoder 1 contains the context and location information of the input sequence. Similarly, the output state of encoder 2 includes the context, previously predicted target token, and intention information. Therefore, with the calculation of attention distribution, the results are determined by the above information.

## 3. Linguistic Model Based on MMI and FPM 

To guarantee the existence of the output sequence, the probability of the predicted target sequence needs to be estimated by the linguistic model. In addition, a linguistic model based on MMI, which can improve the response coherence of the conversational system and reduce the generation of irrelevant responses, is adopted to estimate the probability of the output sequence in the paper [6]. Nevertheless, foolish responses such as “I don’t know” and “what?” are still unavoidable in the process of testing. Therefore, a foolish punishment mechanism (FPM) is added to the linguistic model based on MMI to reduce the number of foolish responses.

(19) $$P(\hat{Y})=\max\text{\{}\log p(\hat{Y}|X)-\lambda \log U(\hat{Y})\text{\}}$$

(20) $$U(\hat{Y})=\prod_{n=1}^{N}{p(\hat{y}}_{n}|{\hat{y}}_{1}{,\hat{y}}_{2},{\hat{y}}_{3},\cdots {\hat{y}}_{n-1})\cdot g(n)$$

(21) $$g(n)=\{\begin{array}{c} \begin{array}{cc} 1 & n\leq \gamma \end{array}\\ \begin{array}{cc} 0 & n>\gamma \end{array} \end{array}$$

where λ is a hyper parameter for the general response punishment; γ is the first token to be punished; and n is the index of the target tokens, which is generated at time n.

The predicted target tokens are punished by calculating the probability of foolish responses Y, which is predicted by the previous output sequence Ŷ. For example, the current target token is predicted based on the previous output sequence as input. Then the target token is compared with the foolish responses. If the predicted target token is similar to the foolish response tokens, then the token is regarded as a foolish target token. According to the comparison results, the probability of the predicted target tokens being foolish response tokens is obtained, and the probability is used as the punishment for foolishness. Ten sequences Y of foolish responses like “I don’t know” and “I have no idea” are manually built, which are often generated by the general model. Although the system generates more total categories of foolish responses than the manually built sequences of foolish responses, these responses will be similar to the established foolish responses. Therefore, the foolish punishment function is defined as

(22) $${R(\hat{Y}}_{n}{=y}_{n}^{\left(k\right)})=\frac{1}{N_{Y}}\{\sum_{y\in Y}\frac{1}{N_{y}}\log p(Y|\hat{Y})\}$$

where N_Y_ is the number of foolish responses; N_y_ is the number of tokens in the foolish responses Y. Meanwhile, the final objective function is defined as

(23) $$P(\hat{Y})=\max\{\log p(\hat{Y}|X)-\lambda_{1}\log U(\hat{Y})-\lambda_{2}R(\hat{Y})\}$$

where λ_1_ and λ_2_ are hyper parameters. Both are set to be equal to 0.25.

In the test, the generative conversational system needs to sample the predicted target tokens to maximize the probability of the output sequence. In addition, the Beam Search algorithm [23] is often adopted. The Beam Search algorithm is a graph-searching algorithm that can quickly find the optimal output sequence. However, the Beam Search algorithm is prone to generating erroneous responses in the sampling process, e.g., the traditional Beam Search algorithm is easily affected by previously sampled tokens and large local probabilities. Moreover, the correct response sequence cannot be produced. Therefore, in this paper, the Diverse Beam Search algorithm [24] is used to predict target tokens, as it is able to improve the diversity of output sequences by sampling on the basis of grouping using the Beam Search algorithm.

## 4. Experiments

### 4.1. Datasets and Training

The OpenSubtitles (OSDb) dataset, an open-domain dataset, is applied in these experiments. The OSDb contains 60M scripted lines spoken by movie characters [25]. 301,000 question–answer pairs are randomly selected, of which 300,000 are used for training and 1000 are sampled for testing. 512 hidden units are adopted for the dual encoder in the model. All embedded vectors have a dimensionality of 512. Meanwhile, the same dimensionality is also adopted for linear layer mapping between the embedded sizes and hidden layers; a learning rate of 0.001 is used. In addition, subsequently, a mini-batch of 256 is used; the filter widths are set to 3 and 5, respectively, and the stacked convolution blocks are set to 3 in both encoders. The model is trained with mini-batches by back-propagation, and the gradient descent optimization (Adam Optimizer) is performed.

### 4.2. Automatic Evaluations

Automatic evaluations of response quality are an open and difficult problem in the conversational field [19,26]. In addition, while there are existing automatic evaluation methods related to machine translation, such as Bilingual Evaluation Understudy (BLEU) and METEOR, these metrics for evaluating conversational system do not correlate strongly with human evaluations, and have been negated by many scholars for the purposes of conversational evaluation [19]. Influenced by the automatic evaluation of multi-turns and response diversity, as proposed by Li [16,27], in which the degree of response diversity is calculated by the number of distinct unigrams in the generated responses, and inspired by conversational targets, the authors propose two automatic evaluation criteria—response diversity and response coherence—in order to indirectly reflect the relationships between system responses and real responses. 

Response Coherence: the proposed measure for evaluating response coherence is to compute the cosine similarity between the question and the system responses based on embedding using the greedy matching method [18]. In other words, the similarities between the question and the responses are calculated by random sampling of samples in the test. In addition, the mean operation is applied to the similarity of the samples. The coherence of question and responses is greater where the similarity is greater.

Response Diversity: Although the method of BLEU [28] is pointed out as being unreasonable for evaluating the coherence between system response and human evaluation [19], the idea behind BLEU is to calculate the similarity between two sequences. Therefore, response diversity is proposed to be calculated by an improved method of BLEU, which evaluates response diversity on the basis of a calculation of candidates for responses, instead of a calculation of system responses and real response [15,16]. Candidates for responses used in the test are generated by the Diverse Beam Search algorithm. The value of BLEU is obtained by pairwise calculation of candidates and averaged by mean operation. Multiple candidates generated each time are defined as a sample. In addition, the means of a sample calculated by the BLEU method are regarded as the response diversity. Samples are sampled randomly, and response diversity is calculated during the test. Response diversity is greater when the similarity is weaker.

Length of the Conversation: Li et al. [16] proposed a method for evaluating the turns of a conversation: a conversation ends when a foolish response like “I don’t know” is generated, or two consecutive responses are highly overlapping. In the test, the above method is adopted to determine the length of a conversation in which eight interactions are defined as one turn. 

### 4.3. Human Evaluation

Although the response quality of the system can be indirectly reflected by the coherence, diversity and length of the conversation, the relationship between system responses and real responses cannot be determined by their simple linear superposition. Therefore, the current popular method of human evaluation is used for comprehensive evaluation.

To improve the quality of human evaluation, 500 data points are randomly collected from the test questions and responses, and the system responses and baseline model responses are labeled by five volunteers. Meanwhile, five-grade interpretation criteria proposed by Zhang et al. [29] are adopted as labeling criteria.

1. It is not fluent or is logically incorrect in responses;
2. The response is fluent, but irrelevant to the question, including irrelevant regular responses;
3. The response is fluent and weakly related to the question, but the response can answer the question;
4. The response is fluent and strongly related to the question;
5. The response is fluent and strongly related to the question. The response is close to human language.

## 5. Results

Four baseline models were used in the experimental comparison, including an attention mechanism-based seq2seq model (LSTM+Attention) [7,22], an attention with intention-based model (LSTM+AWI) [16], an MMI-based AWI model (LSTM+AWI+MMI) [15,16], and an attention mechanism--based CNN model (CNN+Attention) [9,22]. 

In the test sample, 1000 samples were randomly collected in order to calculate the response coherence, response diversity, and length of the conversation. As can be seen from Table 1, SAWI-DCNN except MMI+FPM scored 0.534, 0.670 and 3.25 for coherence, diversity and length of the conversation, respectively. Compared with LSTM+AWI, the structural model of SAWI-DCNN exhibited increases of 14.6%, 3.6% and 10.2%. So SAWI-DCNN showed a certain improvement in structure. SAWI-DCNN scored 0.680, 0.607 and 4.05. Compared with SAWI-DCNN except MMI+FPM, SAWI-DCNN exhibited increases of 27.3%, 9.4% and 24.6%. At the same time, compared with LSTM+AWI+MMI, SAWI-DCNN exhibited increases of 24.7%, 9.7% and 10.7%. Therefore, the joint model showed great improvement in terms of response quality. Samples randomly collected by SAWI-DCNN with the beam size of 6 in the Diverse Beam Search algorithm divided into 3 groups are shown in Table 2. As can be seen from the data, the output system responses are diverse and coherent. In addition, the model trends toward generating short responses. Meanwhile, foolish responses may be produced with an increase in the length of the question.

100 samples were collected randomly by SAWI-DCNN. The results for the percentage of foolish responses are shown in Table 3, where the foolish responses were calculated and classified using the BELU metric. In other words, the BELU value was calculated by the output response and the predefined foolish responses. In addition, when the BELU value was more than 0.5, the response was considered to be a foolish one. As can be seen from the data, compared with SAWI-DCNN without FPM, the joint SAWI-DCNN has a strong inhibitory effect on foolish responses.

The Diverse Beam Search algorithm was used to sample the predicted target tokens and select the candidates with the greatest likelihood probability. Some of the sampling results are shown in Table 4. It can be seen that the SAWI-DCNN trends toward generating high-quality responses, whereas foolish responses like “I don’t know what you are talking about” and “what?” are easily produced by LSTM+Attention and CNN+Attention.

The responses of SAWI-DCNN and the baseline models were sampled randomly and evaluated by humans. The results are shown in Table 5, where the labels (1–5) correspond to the grading against the five-step interpretation criteria. For example, 1 corresponds to the response “It is not fluent or is logically incorrect in responses”, and 2 corresponds to the response “The response is fluent, but irrelevant to the question, including irrelevant regular responses”. Values are the percentage of the response number for the sample collected in each grade. The larger the ratio, the more prone the model is to producing responses with the corresponding feature in the five-step interpretation criteria. The quality of the model can be judged on the basis of the response distribution in the corresponding five-step interpretation criteria, i.e., the higher the quality of model response is, the higher the distribution of grades will tend to be in the responses. The parameter AVE is the average grade of responses, which is calculated on the basis of the corresponding response distribution of the samples and the weights. As can be seen from the data in Table 5, high-grade responses are more easily generated by SAWI-DCNN than by the baseline models. In addition, there is a trend towards high-quality responses being produced with higher average grade scores.

## 6. Conclusions

In this paper, a generative conversational system was investigated based on a structural model and a linguistic model. The structural model was initially established based on the new CNN and strengthened attention with intention. Similarly, the linguistic model was established based on MMI and FPM. Both were combined into the form of a conversational system. Different models were tested and evaluated under automatic evaluation and human evaluation. The results of automatic evaluation were observed and compared in terms of response diversity, response coherence, and length of the conversation. Meanwhile, to the results of the proposed method were also observed and compared based on human evaluation in terms of comprehensive response quality. Finally, by evaluating these comparative results, it can be concluded that the proposed joint model greatly and significantly improves the conversational system. This work paves the way for generative conversational systems, in which the optimal combination of a structural model and a linguistic model is the key to improving the response quality of the system.

## Figures and Tables

**Table 1:** The metric-based evaluation.

Models	Coherence	Diversity	Length
LSTM+Attention	0.371	0.792	2.40
LSTM+AWI	0.466	0.695	2.95
CNN+Attention	0.341	0.801	2.14
LSTM+AWI+MMI	0.545	0.672	3.66
SAWI-DCNN	0.680	0.607	4.05
SAWI-DCNN (except MMI+FMP)	0.534	0.670	3.25

**Table 2:** The responses based on Diverse Beam Search.

Inputs	Responses
What are you doing?	1. I am talking to you.
	2. I am looking for you
	3. I want to talking to you.
	4. I do nothing
	5. I get out of here.
	6. I am not sure.
Wit beyond measure is man’s greatest treasure.	1. Wisdom is the most precious, human beings should have it.
	2. Wisdom is the most precious, human beings should have.
	3. Wit is greatest treasure.
	4. Wit is biggest treasure.
	5. Yes, we should possess greatest treasure.
	6. Yes, we should possess.
It is not our abilities that show what we truly are, it is our choices.	1. Good choice is helpful to us, is not it?
	2. Good choice determines you are.
	3. Abilities can not show we are.
	4. Abilities it are.
	5. I don’t know what you are talking.
	6. I don’t know what you are talking about.

**Table 3:** Foolish responses evaluation (%).

Models	Foolishness
SAWI-DCNN	8%
SAWI-DCNN (except FMP)	26%

**Table 4:** Samples generated by SAWI-DCNN and baseline models on the OSDb.

**#Input**	**What are you doing?**
SAWI-DCNN	I am talking to you
LSTM+Attention	I do thinking.
LSTM+AWI	I want to talk to you
CNN+Attention	I am fun.
LSTM+AWI+MMI	I want to talk to you.
**# Input**	**I mean. I have to talk to you.**
SAWI-DCNN	Come on, let is talking about it.
LSTM+Attention	Ok.
LSTM+AWI	I know you are.
CNN+Attention	I don’t know.
LSTM+AWI+MMI	I mean. He is a good gun.
**# Input**	**Wit beyond measure is man’s greatest treasure.**
SAWI-DCNN	Wisdom is the most precious, human beings should have it.
LSTM+Attention	I don’t know.
LSTM+AWI	Yes. Man has greatest treasure.
CNN+Attention	I don’t know what are you talking about.
LSTM+AWI+MMI	Of course. wit is greatest treasure.
**# Input**	**It is not our abilities that show what we truly are, it is our choices.**
SAWI-DCNN	Good choices are helpful to us, do we?
LSTM+Attention	I don’t know.
LSTM+AWI	We should not choice abilities.
CNN+Attention	I do not know what you are talking.
LSTM+AWI+MMI	We should look at our choices correctly.
**# Input**	**The things we lose have a way of coming back to us in the end, if not always in the way we expect.**
SAWI-DCNN	The things comes back.
LSTM+Attention	I don’t know what you are talking about.
LSTM+AWI	I don’t know what you are talking about.
CNN+Attention	What?
LSTM+AWI+MMI	Yes it is

**Table 5:** Human evaluation of five-grade interpretation criteria (%).

Models	1	2	3	4	5	AVE
LSTM+Attention	17.2	32.7	28.1	17.4	4.6	17.30
LSTM+AWI	12.0	28.7	33.5	20.3	5.5	18.57
CNN+Attention	18.5	30.1	20.5	27.3	3.6	17.84
LSTM+AWI+MMI	12.5	21.8	29.4	25.5	10.8	20.02
SAWI-DCNN	10.6	19.0	19.9	40.3	10.2	21.37

## References

[1] Xing C., Wu W., Wu Y., Liu J., Huang Y., Zhou M., Ma W.Y. (2017). Topic Aware Neural Response Generation. Proceedings of the AAAI’17 Thirty-First AAAI Conference on Artificial Intelligence.

[2] Kaisheng Y., Baolin P., Geoffrey Z., Kam-Fai W. (2016). An Attentional Neural Conversation Model with Improved Specificity. arXiv.

[3] Cao K., Clark S. Latent Variable Dialogue Models and their Diversity. Proceedings of the 15th Conference of the European Chapter of the Association for Computational Linguistics: Short Papers.

[4] Sordoni A., Bengio Y., Vahabi H., Lioma C., Grue Simonsen J., Nie J.Y. A Hierarchical Recurrent Encoder-Decoder for Generative Context-Aware Query Suggestion. Proceedings of the CIKM ’15 24th ACM International on Conference on Information and Knowledge Management.

[5] Serban Iulian V., Sordoni A., Lowe R., Charlin L., Pineau J., Courville A., Bengio Y. (2017). A Hierarchical Latent Variable Encoder-Decoder Model for Generating Dialogues. Proceedings of the Thirty-First AAAI Conference on Artificial Intelligence.

[6] Shen X., Su H., Li Y., Li W., Niu S., Zhao Y., Aizawa A., Long G. (2017). A Conditional Variational Framework for Dialog Generation. Assoc. Comput. Linguist..

[7] Sutskever I., Vinyals O., Le Q.V. (2014). Sequence to Sequence Learning with Neural Networks. Adv. Neural Inf. Process. Syst..

[8] Mou L., Song Y., Yan R., Li G., Zhang L., Jin Z. (2016). Sequence to Backward and Forward Sequence: A Content-Introducing Approach to Generative Short-Text Conversation. Assoc. Comput. Linguist..

[9] Dauphin Y.N., Fan A., Michael A., Grangier D. (2017). Language Modeling with gated convolu-tional networks. Int. Mach. Learn. Soc..

[10] Fuji R., Jiawen D. (2018). Background Knowledge Based Multi-Stream Neural Network for Text Classification. Appl. Sci..

[11] Calvo-Zaragoza J., Francisco J.C., Gabriel V., Ichiro F. (2018). Deep Neural Networks for Document Processing of Music Score Images. Appl. Sci..

[12] Gehring J., Michael A., Grangier D., Yarats D., Dauphin Y.N. (2017). Convolutional Sequence to Sequence Learning. Int. Mach. Learn. Soc..

[13] Zhang Y., Wallace B.C. (2017). A Sensitivity Analysis of (and Practitioners’ Guide to) Convolutional Neural Networks for Sentence Classification.

[14] Gehring J., Michael A., Grangier D., Yaun N.D. (2016). A Convolutional Encoder Model for Neural Machine Translation. ACL.

[15] Jiwei L., Michel G., Brockett C., Jianfeng G., Dolan B. A Diversity-Promoting Objective Function for Neural Conversation Models. Proceedings of the NAACL-HLT 2016.

[16] Li J., Monroe W., Ritter A., Galley M., Jianfeng G., Jurafsky D. Deep Reinforcement Learning for Dialogue Generation. Proceedings of the 2016 Conference on Empirical Methods in Natural Language Processing.

[17] Grosz B. (1986). Attention, Intention, and the Structure of discourse. Comput. Linguist..

[18] Yao K., Zweig G., Peng B. Attention with Intention for a Neural Network Conversation Model. Proceedings of the NIPS 2015 Workshop on Machine Learning for Spoken Language Understanding and Interaction.

[19] Liu C.W., Lowe R., Serban I.V., Noseworthy M., Charlin L., Pineau J. How Not to Evaluate Your Dialogue System: An Empirical Study of Unsupervised Evaluation Metrics for Dialogue Response Generation. Proceedings of the 2016 Conference on Empirical Methods in Natural Language Processing.

[20] Kaiming H., Xiangyu Z., Shaoqing R., Sun J. Deep residual learning for image recognition. Proceedings of the IEEE Computer Society.

[21] Kalchbrenner N., Grefenstette E., Blunsom P. A Convolutional Neural Network for Modelling Sentences. Proceedings of the 52nd Annual Meeting of the Association for Computational Linguistics.

[22] Minh-Thang L., Hieu P., Manning C.D. Effective Approaches to Attentional-Based Neural Machine Translation. Proceedings of the 2015 Conference on Empirical Methods in Natural Language Processing.

[23] Markus F., Yaser A. Beam Search Strategies for Neural Machine Translation. Proceedings of the First Workshop on Neural Machine Translation.

[24] Vigayakumar A.K., Cogswell M., Selvaraju R.R., Sun Q., Stefan L., Crandall D., Batra D. Diverse Beam Search: Decoding Diverse Solutions from Neural Sequence Models. Proceedings of the International Conference on Learning Representations.

[25] Tiedemann J. (2009). News from OPUS—A collection of multilingual parallel corpora with tools and interfaces. Recent Advances in Natural Language Processing.

[26] Pietquin O., Hastie H. (2013). A Survey on Metrics for the Evaluation of User Simulations. Knowl. Eng. Rev..

[27] Galley M., Brockett C., Sordoni A., Ji Y., Auli M., Quirk C., Mitchell M., Gao J., Dolan B. δ BLEU: A discriminative metric for generation tasks with intrinsically diverse targets. Proceedings of the 53rd Annual Meeting of the Association for Computational Linguistics and the 7th International Joint Conference on Natural Language Processing (Short Papers).

[28] Papineni K., Roukos S., Ward T., Zhu W.J. BLEU: A Method for Automatic Evaluation of Machine Translation. Proceedings of the 40th Annual Meeting of the Association for Computational Linguistics.

[29] Zhang H., Lan Y., Guo J., Xu J., Cheng X. Reinforcing coherence for sequence to sequence model in dialogue generation. Proceedings of the International Joint Conferences on Artificial Intelligence.

